# Prophylactic use of incisional negative pressure wound therapy for groin incisions in vascular surgery: randomized clinical trial

**DOI:** 10.1093/bjsopen/zraf059

**Published:** 2025-06-12

**Authors:** Alireza Daryapeyma, Olga Nilsson, Jennifer Pettersson, Otto Stackelberg, Eva Torbjörnsson, Rebecka Hultgren

**Affiliations:** Department of Molecular Medicine and Surgery, Karolinska Institutet, Stockholm, Sweden; Department of Vascular surgery, Karolinska University Hospital, Stockholm, Sweden; Department of Molecular Medicine and Surgery, Karolinska Institutet, Stockholm, Sweden; Department of Vascular surgery, Karolinska University Hospital, Stockholm, Sweden; Liljeholmen University Primary Health Care Centre, Stockholm, Sweden; Department of Clinical Science and Education, Södersjukhuset, Karolinska Institutet, Stockholm, Sweden; Department of Clinical Science and Education, Södersjukhuset, Karolinska Institutet, Stockholm, Sweden; Department of Surgery, Stockholm South General Hospital, Stockholm, Sweden; Department of Molecular Medicine and Surgery, Karolinska Institutet, Stockholm, Sweden; Department of Vascular surgery, Karolinska University Hospital, Stockholm, Sweden

## Abstract

**Background:**

The efficacy of incisional negative pressure wound therapy (iNPWT) in preventing surgical site infections (SSIs) after infrainguinal vascular surgery remains unclear. This study compared iNPWT with standard gauze dressings to determine the effects on SSI incidence and quality of life.

**Methods:**

Patients undergoing infrainguinal vascular surgery were recruited to a regional randomized clinical trial. All patients had a groin incision for either thromboendarterectomy only with a vein or synthetic patch or hybrid procedures with thromboendarterectomy and concurrent endovascular treatment. The control and intervention groups received sterile gauze and iNPWT dressings, respectively. The primary endpoint was the incidence of SSIs within 30 days, measured using the Additional treatment, Serous discharge, Erythema, Purulent exudates, Separation of the deep tissues, Isolation of bacteria and inpatient Stay (ASEPSIS) scoring protocol. Secondary endpoints were scores on the Wound Quality of Life and EQ-5D™ three-level (EQ-5D-3L™) questionnaires.

**Results:**

Of the 123 patients randomized, 109 (89%) completed the study. The incidence of SSI was similar in the intervention and control groups (15% *versus* 25%, respectively; *P* = 0.340), but was higher in patients with a body mass index (BMI) ≥ 25 kg/m^2^ than in those with a BMI < 25 kg/m^2^ (52% *versus* 21%, respectively). Wound Quality of Life mean scores were similar in the control and intervention groups (10.60 *versus* 12.85, respectively; *P* = 0.322); however, the intervention group reported a larger negative impact on everyday life than the control group (7.40 *versus* 4.91 for the everyday life domain, respectively; *P* = 0.048). There were no significant differences between the two groups in EQ-5D-3L™ scores.

**Conclusion:**

The similar distribution of SSIs regardless of the type of wound dressing, in addition to the negative impact on everyday life, does not support the general use of iNPWT in this or similar patient cohorts. The unique aspect of this trial is the patient perspective on the use of iNPWT, revealing a divergence between the views of patients and healthcare providers regarding optimal wound care. In the setting of individualized care, there may be a place for iNPWT in selected patients with a high BMI and excessive perioperative bleeding, which are associated with a higher SSI risk.

**Registration number:**

NCT03395613 (https://clinicaltrials.gov).

## Teaser

In this randomized trial of 109 patients in Sweden, incisional negative pressure wound therapy (iNPWT) was not associated with a decreased incidence of surgical site infection after surgery. Patients receiving iNPWT experienced a larger negative impact on everyday life than those treated with standard gauze dressings. The results support the use of iNPWT in selected patients, and point to the importance of including patient aspects when considering wound treatment.

## Introduction

Surgical site infections (SSIs) after infrainguinal procedures are among the most common healthcare-associated infections in vascular surgery, with a reported incidence of up to 30%^1–4^. Endogenous contamination from the patient's skin flora is the most common cause of SSIs^5,6^. In infrainguinal procedures, the general risk of SSIs is compounded by the proximity of surgical incisions to the urogenital tract and the perineum, areas with high microbial load^7^. SSI development is contingent on a multifactorial process driven by coexisting factors such as patient, surgical, and microbial characteristics. Some of the major patient characteristics that can influence the course and outcome of SSIs are age, smoking, and co-morbidities such as diabetes and obesity^8–10^. Among the surgical characteristics relevant to SSIs are the duration of surgery, reoperation, type of wound, and the need for blood transfusion^11–13^.

SSIs after infrainguinal vascular surgery may lead to increased patient suffering and decreased quality of life (QoL), a prolonged hospital stay, increased healthcare costs, and limb amputation^14–16^. Various strategies have been tested, with different degrees of success, to reduce the incidence of SSIs. These strategies include preoperative preparatory procedures (such as antiseptic skin preparations and antibiotic prophylaxis), perioperative interventions (such as antimicrobial sutures), and postoperative measures (such as different types of wound dressing)^17–19^. Given the incidence and consequences of SSIs after infrainguinal vascular procedures, any appreciable decrease in the occurrence of these complications could benefit patients and healthcare facilities.

The application of incisional negative pressure wound therapy (iNPWT) to closed surgical wounds is used as a preventive measure against infection or as treatment in the event of manifest infection. iNPWT builds on the concept of open wound treatment with negative pressure wound therapy and can lead to reduced seroma formation, a reduced risk of wound rupture by apposing wound edges, increased capillary circulation, and cell proliferation, resulting in better wound healing and a reduction in SSI^20–22^.

Various vacuum-assisted devices are available for treating chronic and infected wounds, as well as open surgical wounds. Although the use of iNPWT purportedly decreases the incidence of SSIs after infrainguinal vascular surgery, previous studies^20,21,23–27^ have been performed on heterogeneous populations and reported somewhat divergent results. However, the evidence based on data from randomized clinical trials (RCTs) investigating the effectiveness of this prophylactic measure remains equivocal and high-quality RCTs are warranted^28^. Furthermore, iNPWT requires the attachment of a pump to the wound dressing for an extended period of time, and data pertaining to patient experience and QoL are lacking.

The primary aim of the present study was to evaluate the effect of iNPWT compared with standard of care on the incidence of SSIs in closed, single-incision surgical wounds after elective open infrainguinal vascular procedures. The secondary aim was to investigate the effect of iNPWT on QoL.

## Methods

### Design

This was an RCT of consecutively recruited patients.

### Outcomes

The primary outcome measure was defined as SSI within 30 days of surgery and classified with the validated wound scoring protocol Additional treatment, Serous discharge, Erythema, Purulent exudates, Separation of the deep tissues, Isolation of bacteria and inpatient Stay (ASEPSIS). The secondary outcome measure was the impact of iNPWT on patient-reported QoL parameters, assessed using the Wound Quality of Life (Wound-QoL) questionnaire 7 days after surgery and the EQ-5D^™^ three-level (EQ-5D-3L™) questionnaire (EuroQoL Group, Rotterdam, the Netherlands) 30 days after surgery.

### Population

**Fig. 1:**
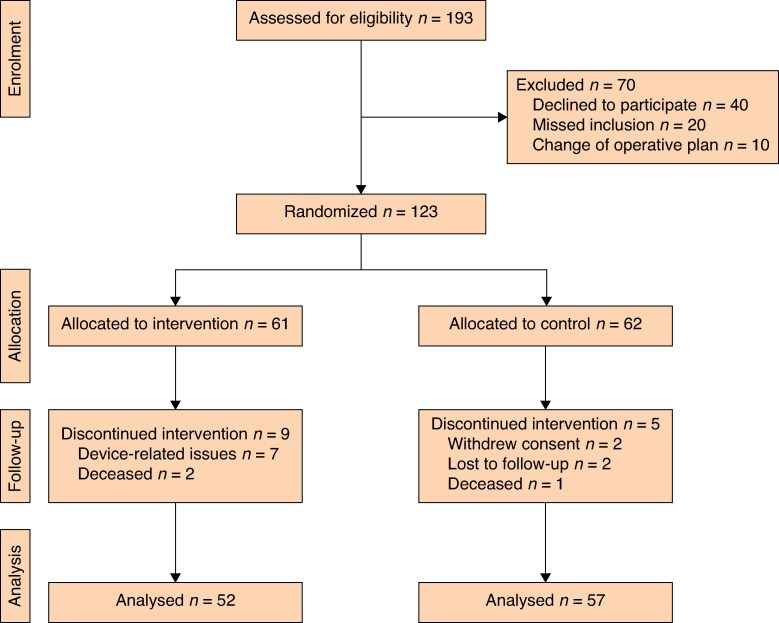
CONSORT diagram outlining study procedures

All patients meeting the inclusion criteria at Karolinska University Hospital and Stockholm South General Hospital between 2018 and 2023 were asked to join the study, and to provide written consent after receiving oral and written information about the study (*Fig. 1*). These centres cover all vascular surgical procedures in a region with a population of 2.4 million inhabitants. Inclusion criteria were patient age > 18 years and a plan for elective, open, infrainguinal vascular surgery for peripheral arterial disease by thromboendarterectomy (TEA) with a vein or synthetic patch or hybrid procedures with TEA and concurrent endovascular treatment. Patients who were considered emergency cases, were undergoing isolated endovascular interventions, had current infection in the groin region at the time of surgery, were sensitive to iNPWT material, and who declined to participate were excluded from the study. Patients treated for iatrogenic arterial lesions after other procedures, such as coronary angiography, were also included in the study. The remaining patients had atherosclerotic lesions in the femoral artery, suitable for TEA. The indication for treatment was categorized as claudication, ischaemic rest pain, ischaemic ulcer/gangrene and iatrogenic lesion.

### Measurement and instruments

The ASEPSIS protocol was used to assess the occurrence of SSIs^29^. This protocol assesses wound length, signs of infection (for example serous or purulent exudate, erythema, and/or separation of deep tissue), the eventual use of antibiotics, surgical revision of the wound, bacterial culture results, and length of hospital stay^29^. The maximum ASEPSIS score is 100, with scores of 0–10 indicating satisfactory healing, scores of 11–20 indicating a disturbance to wound healing, scores of 21–30 indicating minor wound infection, scores of 31–40 indicating moderate wound infection, and scores > 40 indicating severe wound infection.

QoL was measured before and after surgery using the EQ-5D-3L™. The EQ-5D-3L™ consists of two parts. The first covers five dimensions of QoL (mobility, self-care, ability to undertake usual activities, pain/discomfort, and anxiety/depression), with lower scores indicating better QoL^30^. The second part consists of a visual analogue scale (VAS), which the respondents use to rate their perceived health by marking a line between 0 and 100, with 0 indicating the worst possible health. The Wound Quality of Life (Wound-QoL) is a wound-specific 17-item questionnaire that generates 3 subscales (body, psyche, and everyday life) and a total score^31,32^. Higher scores on the Wound-QoL instrument indicate a larger negative impact on a patient's wound-related QoL.

All instruments used in this study have been translated, validated, and adapted to Swedish conditions.

### Randomization and blinding

Once the skin incision had been closed in the operating room, patients were randomized to either the intervention group, receiving the iNPWT device Prevena Plus™ (3M Canada, London, ON, Canada), or the control group, receiving sterile OPSITE Post-Op Visible (Smith & Nephew, Shanghai, China) wound dressing. Randomization was conducted by the Karolinska Trial Alliance, generating a scheme by using the online system http://www.randomization.com/, with a block size of ten patients at a time. The surgeons performing the procedure were blinded to the type of dressing to be applied at the end of the operation. The patients and investigators evaluating the outcome were not blinded to the intervention. In the event of a bilateral groin incision, the patient's right groin was randomized and included in the study.

### Preoperative and perioperative procedures

All patients underwent standard-of-care preoperative preparations (hair removal, 2 body washes with 4% chlorhexidine gluconate). In addition, all patients received perioperative standard care (3 doses of antibiotic prophylaxis with cloxacillin 2 g intravenously, 1 before the surgery and 2 doses after surgery on the day of operation). Groin incisions were closed with subcutaneous and intracutaneous monofilament absorbable sutures.

Both the iNPWT and sterile wound dressings were applied in the operating room and remained on the patients until postoperative day 7, adhering to the instructions for use. The iNPWT device was set to the standard continuous pressure of −125 mmHg. If the vacuum seal was broken for any reason, the iNPWT device was removed and a sterile gauze dressing was applied to the incision instead. The sterile wound dressing could be changed by a research nurse or attending surgeon, if needed, during the 7-day period. After the initial 7 days, patients in both groups were supplied with sterile gauze dressings for an additional 6 days. All handling of wound dressings was performed under sterile conditions, both in hospital and via primary healthcare facilities after discharge.

### Data collection

The groin incisions were examined, evaluated, and categorized by a qualified research nurse 7 and 30 days after surgery. At follow-up, the ASEPSIS wound scoring protocol was used. Blinded assessments were not possible for logistic reasons. If the ASEPSIS score at any one of the two follow-up visits was ≥ 11 points, wound cultures were obtained to identify the infectious agent positively where possible.

Data regarding adverse events (AEs) directly related to the iNPWT dressing and leading to discontinuation of treatment, SSIs within 30 days of surgery, and antibiotic prescriptions for skin and soft tissue infections within 30 days after surgery were collected.

All patients were asked to respond to the EQ-5D-3L™ questionnaire at the time of study inclusion and at the 1-month follow-up visit. Patients were asked to complete the Wound-QoL questionnaire at the 1-week follow-up. Data regarding patient characteristics and demographics, as well as co-morbidities, type of surgical intervention, randomization, and outcome parameters, were obtained from patients’ electronic health records. The collected data were compiled and anonymized after acquisition.

### AEs

An AE was defined as any medical occurrence in a patient who received the medical device, which does not necessarily have a causal relationship to the treatment. All AEs were recorded in the medical records. A serious AE was any adverse device experience that resulted in any of the following outcomes: death, life-threatening AE, persistent or significant disability/incapacity, in-patient hospitalization, or prolonged hospitalization.

### Statistical analysis

Continuous variables are presented as mean(standard deviation, s.d.), and categorical variables as frequency counts and percentages. The Shapiro–Wilk test was used to determine the normality of data distribution. Data were analysed using independent *t* tests for continuous variables and the Mann–Whitney *U* test for non-parametric data. Fisher's exact test was used for comparisons of categorical variables. Comparative analysis was performed with regard to risk factors and outcomes, both primary and secondary, as defined previously. Statistical analyses were undertaken using SPSS^®^ for Mac^®^, version 26 (IBM, Armonk, NY, USA). Patients were excluded from the study cohort if the iNPWT dressing was removed within the first 7 days after surgery.

The sample size estimation was based on an incidence of SSIs in the groin region of up to 33%^33,34^. Previous studies^20,21^ have reported a reduction in the incidence of SSIs by 50% with a negative pressure incision management system, but used different inclusion criteria and heterogeneous populations. In the present study, the sample size calculations for a binary outcome, superiority outcome with an α level of 0.05 for 80% power, an estimated incidence reduction from 30 to 10% using iNPWT in a reasonably homogenous cohort, and an estimated attrition rate of 10%, yielded a sample size of 59 patients in each arm, with a total sample size of 118 patients to be recruited^35^.

### Ethical considerations

This study was performed in accordance with the Declaration of Helsinki, as revised at the 64th WMA General Assembly in Fortaleza, Brazil, October 2013. The study was approved by the Swedish Ethical Review Authority (2017/195-31/1). Before enrolment, all patients received written and oral information about the study, and written informed consent was obtained from all participants. The study is registered with ClinicalTrials.gov (NCT03395613).

## Results

### Patient characteristics

Across the 2 institutions, 193 consecutive patients were assessed for study eligibility, with 123 being randomized (*Fig. 1*). After randomization, 9 patients in the intervention group and 5 patients in the control group withdrew from the study, resulting in 52 patients in the intervention group and 57 in the control group completing the study. Of the nine participants who chose to withdraw from the study, seven stated device-related issues as their primary reason for discontinuation. For all patients, the mean(s.d.) age at the time of primary surgery was 73.9(8.0) years and most (68%) were men. Sociodemographic variables, co-morbidities, and other risk factors were evenly distributed between the two treatment groups (*Table 1*). Perioperative variables, such as blood loss and duration of operation, were similar between the treatment groups (*Table 1*).

**Table 1:** Characteristics of patients in the control and intervention groups

	Control (*n* = 57)	Intervention (*n* = 52)	P*
**Demographic variables**			
Age (years), mean(s.d.)	75.3(7.0)	72.6(8.7)	0.066†
Sex			0.909
Male	36 (63%)	38 (73%)	
Female	21 (37%)	14 (27%)	
**Medical history**			
Hypertension	48 (83%)	41 (79%)	0.931
Diabetes	23 (40%)	17 (33%)	0.298
Cardiac disease	26 (46%)	21 (40%)	0.427
COPD	5 (9%)	6 (12%)	0.705
Renal impairment	3 (5%)	4 (8%)	0.661
TIA/stroke	10 (17%)	11 (21%)	0.741
Steroid treatment	6 (11%)	7 (13%)	0.838
Never smoked	5 (9%)	4 (8%)	0.115
BMI (kg/m^2^), mean(s.d.)	26.4(4.5)	25.1(3.3)	0.100†
**Indication for surgery**			
Iatrogenic groin lesions	0	2 (4%)	0.174
Claudication	36 (63%)	37 (71%)	
Ischaemic rest pain	9 (16%)	7 (13%)	
Ischaemic ulcer/gangrene	12 (21%)	6 (12%)	
**Type of operation**			
TEA	55 (96%)	47 (90%)	0.255
Hybrid	2 (4%)	5 (10%)	
**Perioperative variables**			
Blood loss (ml), mean(s.d.)	327(579)	267(299)	0.521†
Duration of operation (min), mean(s.d.)	193(61.7)	182(53.4)	0.308†

The control group was treated with standard gauze dressings; the intervention group received incisional negative pressure wound therapy. *χ^2^ test or Fisher’s exact test, except †analysed with independent *t* test. s.d., Standard deviation; COPD, chronic obstructive pulmonary disease; TIA, transient ischaemic attack; BMI, body mass index; TEA, thromboendarterectomy; min, minute.

### SSI

The overall incidence of SSIs within 30 days of surgery was 20.2% (22 of 109 patients). There was no difference in the incidence of SSIs between the control and intervention treatment groups (14 (25%) *versus* 8 (15%), respectively) (*Table 2*).

**Table 2:** Incidence of SSIs in the control and intervention groups

	Control group (*n* = 57)		Intervention group (*n* = 52)		P*
	SSI (*n* = 14)	No SSI (*n* = 44)	SSI (*n* = 8)	No SSI (*n* = 44)	
SSIs within 30 days	14 (25%)		8 (15%)		0.340
**Medical history**					
Diabetes	7 (54%)	16 (36%)	4 (50%)	12 (27%)	0.776
BMI ≥ 25 kg/m^2^	10 (77%)	23 (52%)	6 (75%)	21 (48%)	1.000
**Perioperative variables**					
Blood loss (ml), mean(s.d.)	568(1033)	235(223)	470(515)	228(231)	0.049†
Duration of operation (min), mean(s.d.)	212(85)	187(52)	210(45)	177(54)	0.127†
**Postoperative antibiotic treatment**					
SSI	11 (85%)	0 (0%)	7 (87%)	0 (%)	0.186
Other indication	3 (23%)	3 (7%)	1 (12%)	4 (9%)	0.186

Subgroup analyses were performed according to the presence of diabetes, body mass index (BMI), and perioperative variables. The number of patients in each group with a prolonged antibiotic regimen after the standard prophylaxis is reported. *χ^2^ test or Fisher’s exact test, except †analysed with Mann–Whitney *U* test. SSI, surgical site infection; s.d., standard deviation; min, minute.

The proportion of patients with a high (≥ 25 kg/m^2^) body mass index (BMI) was more than double among those who developed SSIs, in both the control and intervention groups (*Table 2*). Among those who did not develop SSIs, a similar proportion in the control and intervention groups had a high (≥ 25 kg/m^2^) BMI. In the total cohort, perioperative blood loss was significantly higher among those who developed SSIs than among those who did not (535 *versus* 231 ml, respectively; *P* = 0.069). This difference in blood loss in those with and without SSIs was also apparent in the control (568 *versus* 235 ml, respectively) and intervention (470 *versus* 228 ml, respectively; *P* = 0.049) groups separately. There was no significant difference in the duration of the surgery according to SSI development between the treatment groups.

### Cultures and antibiotics

In the intervention group, 12 patients (23%) received postoperative antibiotic treatment, 7 (13%) for SSIs and 5 (10%) for other infections (*Table 2*). In the control group, 17 patients (30%) received postoperative antibiotic treatment, 11 (19%) for SSIs and 6 (11%) for other infections, such as urinary tract infections.

Culture samples were taken from the surgical site from 14 patients (ASEPSIS score ≥ 11 points); 13 of these samples yielded positive results for various infectious agents. In the intervention group, there were six positive cultures from seven confirmed cases of SSI, which included four patients with *Staphylococcus aureus* infection and two with *Enterobacteria* and *Enterococcus faecalis* infection. In the control group, there were 7 positive cultures from 13 confirmed cases of SSI: 1 for *Pseudomonas aeruginosa*, 1 for Group B *Streptococcus*, 1 for Group C and G *Streptococcus*, 1 for Enterobacterales, 1 for Gram-negative mixed flora, one for *S. aureus* and Enterobacterales, and 1 for *Cutibacterium* and *Finegoldia magna*.

### Patient-reported outcomes

**Fig. 2:**
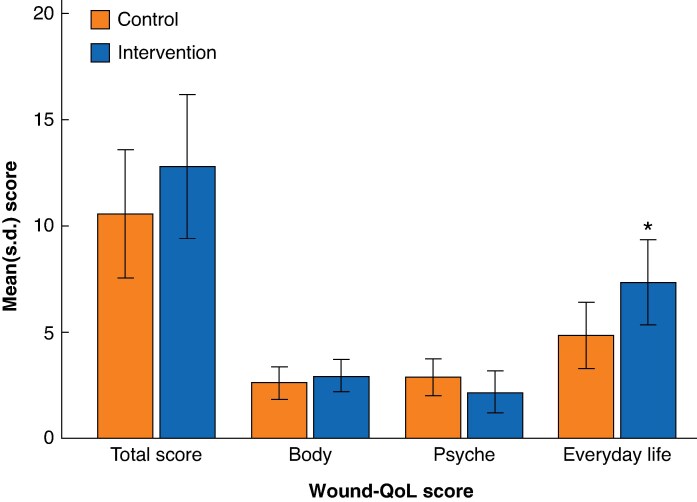
Total Wound-QoL scores across three domains (body, psyche, and everyday life) for the control and intervention groups 1 week after surgery Values are mean(s.d.). Wound-QoL, Wound-Quality of Life. *P = 0.048 *versus* control group; there were no significant differences between the control and intervention groups for the total Wound-QoL score or scores on the body and psyche domains (*P* = 0.322, *P* = 0.505, and *P* = 0.320, respectively) (Analysed with Mann–Whitney *U* test).

At the 1-week follow-up, there were no significant differences in patient-reported Wound-QoL total mean scores between the control and intervention groups (10.60 *versus* 12.85, respectively; *P* = 0.322) (*Table 3* and *Fig. 2*). There were also no significant differences between the two groups for scores on the body and psyche domains of the Wound-QoL. However, the intervention group reported a larger negative impact in the everyday life domain of the Wound-QoL than the control group (7.40 *versus* 4.91, respectively; *P* = 0.048).

**Table 3:** Wound-QoL scores by treatment group reported 1 week after surgery

	Control (*n* = 53)	Intervention (*n* = 52)	P*
Wound-QoL total score, mean(s.d.)	10.60(10.88)	12.85(12.15)	0.322
**Wound-QoL domain score, mean(s.d.)**			
Body	2.64(2.78)	3.99(2.71)	0.505
Psyche	2.91(3.19)	2.25(3.52)	0.320
Everyday life	4.91(5.58)	7.40(7.11)	0.048

*Analysed with Mann–Whitney *U* test. Wound-QoL, Wound Quality of Life; s.d., standard deviation.

There were no significant differences between the control and intervention groups in EQ-5D-3L™ mean scores at baseline or at follow-up (*Table 4*). Improvements were seen in both the EQ-5D-3L™ and EQ-VAS from baseline to follow-up in both treatment groups. When analysing changes from baseline to the 1-month follow-up, there were no significant differences between the control and intervention groups in EQ-5D-3L™ mean scores (mean difference −0.78 *versus* −0.71, respectively; *P* = 0.870) or EQ-VAS scores (11.13 *versus* 11.30, respectively; *P* = 0.967) (*Table 4*).

**Table 4:** Self-reported EQ-5D-3L™ scores by treatment group at baseline and at 1-month follow-up after surgery

	EQ-5D-3L™ score		Mean difference between groups*	*P*†
	Control (*n* = 50)	Intervention (*n* = 48)		
**EQ-5D-3L™ total score**				
Baseline	8.33(2.03)	7.98(1.51)	0.35 (−0.35, 1.06)	0.316
1-month follow-up	7.50(2.44)	7.18(2.23)	0.32 (0.59, 1.24)	0.485
Mean change	−0.78(2.35)	−0.71(1.98)	−0.07 (−0.94, 0.80)	0.870
**EQ-VAS**				
Baseline	59.02(18.87)	59.93(19.11)	0.91 (8.83, 7.02)	0.820
1-month follow-up	69.56(20.60)	71.25(21.21)	−1.69 (−10.28, 6.90)	0.697
Mean change	11.13(20.14)	11.30(17.55)	−0.17 (−8.36, 8.02)	0.967

Values are mean(standard deviation) unless otherwise indicated; *values in parentheses are 95% confidence intervals; †analysed with Mann–Whitney *U* test. The control group was treated with standard gauze dressings; the intervention group received incisional negative pressure wound therapy. EQ-5D-3L™, EQ-5D™ three-level; EQ-VAS, EuroQol visual analogue scale.

## Discussion

This RCT provides robust data on the effect of iNPWT on the prevention of SSIs after infrainguinal vascular surgery, showing that the anticipated reduction in the rate of SSIs was not reached. Furthermore, the larger negative wound-related impact on patients’ everyday life in the iNPWT cohort highlights a novel dimension to consider when choosing treatment strategies for postoperative wound care.

In recent years, a number of RCTs and meta-analyses designs^1–3,5,36–38^ concerning the effects of iNPWT on SSIs have been published, commonly highlighting the heterogeneity of study. One of the most recent meta-analyses by Groenen *et al.*^38^ showed that iNPWT is effective in reducing SSIs, but that meta-analysis included only six studies based on vascular data, with most data stemming from abdominal, orthopaedic/trauma, and obstetric surgeries. The vascular data are further hampered by the heterogeneity of the vascular procedures included. There is also a paucity of data regarding adverse effects of iNPWT, as well as patient-related outcomes. The present study was conceived to try to address some of these issues.

An innovative aspect of this trial was the inclusion of patients’ perspectives on the use of iNPWT as an adjunct to the evaluation of SSI prevention. Although there was a non-significant tendency towards reduced SSI incidence in the intervention group, there is now mounting evidence warranting the restricted use of iNPWT in infrainguinal vascular surgery. This trial purposely only included patients undergoing an open vascular procedure limited to the groin and, as such, represents a highly homogeneous and standardized surgical trauma to a specific anatomical area. The chosen cohort contrasts the present study from several previous studies^27,34,37,39^ that included a more diverse range of vascular procedures.

SSIs are feared complications after vascular surgery and occur at an alarmingly high rate^33,40,41^. The overall incidence of SSIs of 20.2% in the present study falls within the lower range of previously reported rates of SSIs after lower extremity vascular surgery^33,34,42^. The absence of a preventive effect conferred by iNPWT in this study, along with a recently published RCT using PICO™ (Smith+Nephew, Hull, UK), a similar iNPWT system^37^ that included lower extremity bypass surgery, point to the underlying complexity of SSI prevention and the ambiguous role of iNPWT in this respect. The relatively low incidence of SSIs in the present study may be attributed to a number of factors, of which patient selection and the homogeneous nature of the surgical intervention (> 90% endarterectomies) may be considered consequential. A confounding factor in any study investigating the rate of lower extremity SSIs in vascular patients is the suspected effect of long-term preoperative antibiotic usage due to chronic ulceration. The strict inclusion criteria regarding the type of surgery in the present study helped mitigate the effect of this particular confounding factor. This may be further explained by the practice in the region whereby patients with hard-to-heal lower extremity wounds are seldom treated with an isolated groin procedure. The separate analysis of postoperative antibiotic prescriptions relevant to SSI treatment, which mirrored the stated rate of SSIs, adds further validity to the SSI incidence reported in this study.

Diabetes and obesity are known risk factors for postoperative wound complications^43^. Although this data set is too small for such subgroup analyses, an indication of such associations can be found. Patients with a high (≥ 25 kg/m^2^) BMI were over-represented in the group that developed SSIs, in both the control and intervention groups. This association was described in a recent RCT^44^, and indicates that the use of iNPWT may be of value in selected patient groups, such as those with a BMI ≥ 25 kg/m^2^. Furthermore, perioperative blood loss was greater among those who developed SSIs, which is in line with a recently published meta-analysis^45^. In a study^46^ of cardiothoracic procedures, blood loss > 100 ml was identified as an independent risk factor for SSIs. Although no exact threshold for perioperative blood loss has been established in vascular surgery procedures, the present results support the use of iNPWT in patients who experience excessive perioperative bleeding.

This trial included a wound-related patient-reported outcome, namely the Wound-QoL, which was used to assess aspects of QoL related to participants’ surgical wounds. This instrument was originally validated for hard-to-heal wounds, but was chosen for the present study because it was the only validated wound-specific QoL instrument available in Swedish. Within the first week after surgery, patients with iNPWT experienced a larger negative impact on their everyday QoL. The effect of the iNPWT treatment and device may not be negligible, given that seven of the nine patients who withdrew from the intervention group cited device-related issues as a major factor for terminating their participation. In a qualitative study of patients with negative pressure wound therapy, technical responsibility for the device and the sense of being physically limited by it were described as burdens^47^. Termination of iNPWT treatment necessitates premature exposure and a change of dressing of the surgical wound, which may, in turn, increase the risk of SSI development. The cost-effectiveness of iNPWT was not assessed in the present study but, given the almost 10% rate of discontinuation in the intervention group and the consequent need for extra healthcare contacts, this aspect warrants inclusion in future studies.

The intentionally restrictive inclusion criteria, which aimed to provide a homogeneous yet generalizable cohort of patients undergoing vascular treatment, together with the inclusion of patient-reported outcome measures may be considered the main strengths of this RCT. The protracted course of the study was influenced by logistical limitations owing to the relocation of one of the recruiting hospitals and the COVID-19 pandemic. The study also excluded patients who did not fulfil their assigned treatment during the first study week. The nature of the study design did not enable blinding of patients or staff after treatment allocation; this may have led to detection bias in the assessments of treatment effects. Attrition rates were slightly higher in the intervention group because participants withdrew from the study owing to device-related issues. The Wound-QoL instrument has not been validated for surgical wounds but was chosen for the present study owing to the absence of other QoL instruments addressing this cohort of patients. This may have affected the validity of the results reported.

The similar distribution of SSIs regardless of the type of wound dressing, along with the negative impact on everyday life, does not support the general use of iNPWT in this or similar patient cohorts. The unique aspect of this trial is the inclusion of patients’ perspectives on the use of iNPWT, revealing a divergence between the views of patients and healthcare providers regarding optimal wound care. In the setting of individualized care, there may be a place for iNPWT in selected patients with a high BMI and excessive perioperative bleeding, which are associated with a higher risk of SSIs.

## Supplementary Material

zraf059_Supplementary_Data

## Data Availability

The data that support the findings of this study are available from the corresponding author upon reasonable request. The data are not publicly available because they contain information that could compromise participant privacy.
