# Supplementary material for: Prophylactic use of incisional negative pressure wound therapy for groin incisions in vascular surgery: randomized clinical trial
Source: BJS Open. 2025 Jun 12;9(3):zraf059. doi: 10.1093/bjsopen/zraf059 (PMC12158596; doi:10.1093/bjsopen/zraf059)
Supplement: zraf059_Supplementary_Data [file zraf059_supplementary_data.zip › Study protocol.pdf]

Prophylactic use of a Negative Pressure Incision Management System in Infrainguinal Vascular Surgery – A Multicentre Randomized Controlled Trial

Background:

Postoperative infections in vascular surgery represent a substantial burden of disease and have consequences for both the individual and the community.[1-3] Surgical site infections (SSI) are the most common of healthcare associated infections in vascular surgery patients treated with infrainguinal procedures.[4] The development of SSI is contingent on the interplay among three factors: microbial, patient and surgical characteristics. Endogenous contamination from the patient's skin flora is the most common cause of surgical wound contamination. Other microbial related factors include the bacterial load and virulence.[5] Inclusion of prosthetic materials increases the susceptibility of the wound to infection as the number of organisms needed to cause an infection decrease.[6] The virulence of the organisms is dependent on intrinsic factors such as, the ability to bind to tissues and protect themselves from the onslaught of the immune system.[7] The patient characteristics that can influence the emergence and outcome of SSI include age, comorbidities e.g. diabetes mellitus and obesity and concomitant medications such as immunosuppressants.[8,9] The surgical characteristics relevant to SSI are length of surgery, type of wound and the need for blood transfusion.[10, 11]

The use of artificial vascular prostheses accentuates the risks of SSI and deep groin infections, which can lead to grave consequences. Infection in a prosthetic vascular graft is a serious complication which is unfortunately difficult to detect at an early stage and often leads to major morbidity.

Previous studies have reported varying rates of postoperative SSI after infrainguinal surgery with some reporting rates of up to 30%.[12] Various strategies, ranging from preoperative preparatory procedures such as hair removal and antiseptic skin preparations to peroperative interventions such as the use of antimicrobial sutures and carbon dioxide insufflation of the surgical wound to postoperative measures regarding antibiotic prophylaxis and type of wound dressing, have been tested with varying degrees of success in reducing postoperative SSI.[13-15]

Negative pressure wound therapy (NPWT) has been used in treating postoperative SSI with encouraging results and is now an accepted element in the arsenal of tools for treating these complications.[15, 16] The concept of applying NPWT to closed surgical wounds as a preventive measure is relatively new but draws on the inherent properties of the method which could theoretically lead to reduced seroma formation, wound dehiscence, increased capillary circulation and consequently better wound healing and fewer SSI.[17, 18]

Given the incidence and the consequences of SSI in infrainguinal vascular procedures any appreciable decrease in the burden of these complications could have profound benefits for patients and healthcare facilities. As to date there are few published data from randomized controlled trials investigating the effectiveness of this prophylactic measure.

The aim of this study is to assess the effect of NPWT applied to closed surgical wounds directly after skin closure compared to standard sterile gauze dressing in reducing the incidence and severity of postoperative SSI in infrainguinal surgical wounds in patients undergoing elective, open vascular surgery.

Study objectives and hypotheses: To assess the effect of NPWT on closed surgical incisions on reducing the incidence of postoperative SSI.

-To evaluate the effect of NPWT on closed surgical incisions in reducing the severity of postoperative SSI.

-To evaluate the impact of NPWT on the patients' functionality and quality of life

-To analyse the cost effectiveness of NPWT as compared to standard treatment

Study design: Multi centre, population-based, prospective, randomized, controlled, open-label trial of consecutively recruited patients.

Primary outcome measure: Postoperative SSI within 30 day, classified using the scoring method ASEPSIS (Additional treatment, Serous discharge, Erythema, Purulent exudates, Separation of the deep tissues, Isolation of bacteria and inpatient Stay) and graded using the Samson classification (Grade I-V), confirmed by positive identification of the infectious agent.

Secondary outcome measures:

- Postoperative SSI within 90 days, classified using the scoring method ASEPSIS (Additional treatment, Serous discharge, Erythema, Purulent exudates, Separation of the deep tissues, Isolation of bacteria and inpatient Stay) and graded using the Samson classification (Grade I-V), confirmed by positive identification of the infectious agent.
- Antibiotic prescriptions for skin and soft tissue infections within 90 days postoperatively.
- Postoperative SSI within 90 days requiring surgical revision.
- Adverse events directly related the NPWT dressing leading discontinuation of treatment.
- Major lower limb amputation and/or mortality within 90 days postoperatively.
- Changes in reported functionality and quality of life during the 30 day postoperative period.
- Assessment of healthcare related costs within 30 days postoperatively.

Inclusion criteria:

1. Patients older than 18 years.
2. The clinical criterion for study entry is elective open infrainguinal vascular surgery for peripheral arterial disease: i. Thrombendarterectomy (TEA); ii. Thrombectomy with vein or synthetic patch
3. Patients on whom complete seal of NPWT can be maintained during the first 24 hours after operation.

Exclusion criteria:

1. All emergency cases.
2. Infrainguinal endovascular procedures
3. Pre-existing groin infections.
4. Sensitivity/allergy to materials used in NPWT dressing.
5. Patients on whom an adequate and complete seal of NPWT dressing cannot be obtained and/or maintained.
6. Unwillingness to participate.

Intervention and Surveillance: The groin incisions in the intervention group will have NPWT dressing, applied under sterile conditions in the operating room. The NPWT will remain on the patient until postoperative day 7 or earlier if the vacuum seal is broken for any reason. If the NPWT is removed earlier than 7 days, a sterile gauze dressing will be applied to the incision.The control group will receive a sterile gauze dressing, applied under sterile conditions in the operating room. The dressing can, if needed be changed under sterile conditions at the ward on the second postoperative day. The dressing will remain on the patients until postoperative day 7. The dressing may be changed (by a research nurse or the attending surgeon), under sterile conditions if needed during the 7-day period. After the initial 7-day period, patients in both groups will be supplied with sterile gauze dressings for an additional 7 days during which dressing changes may be carried out under sterile conditions. All groin incisions will be examined, evaluated and categorized by a qualified research nurse or the attending surgeon who will be blinded to the initial treatment modality, on the seventh postoperative day, 30 days postoperative and again after three months. Wound cultures will be taken (when possible), if the ASEPSIS score at any one of the three examinations is equal to or exceeds 21 points. Preoperative preparations will be the same for both groups. All patients

will receive three doses (one pre- and two postoperative) of antibiotic prophylaxis on the day of the operation. Groin incisions will be closed with subcutaneous and intracutaneous monofilament absorbable sutures. All patients will be asked to respond to the 36-Item Short Form Health Survey (SF-36) and the European Quality of life – Five dimensions (EQ-5D) questionnaires at the time of inclusion and at the one month follow up visit. The Wound QoL (Swedish) questionnaire will be presented to the patients at the one-week follow up.

Adverse events: An adverse event (AE) is defined as any untoward medical occurrence in a patient administered the medical device which does not necessarily have a causal relationship to the treatment. All AEs will be recorded in the medical records. A serious adverse event (SAE) is any adverse device experience that results in any of the following outcomes:

- Death
- Life-threatening AE
- Persistent or significant disability/incapacity
- Requires in-patient hospitalization or prolongs hospitalization

***Specific to this study*:**

  - Death due to procedural complications
  - Vascular complications
  - Severe or life-threatening bleeding
  - Limb loss
  - TIA/stroke or myocardial infarction
  - Allergic skin reactions
  - Device related skin damage e.g. abrasions, blisters or wounds

Randomization and blinding: Randomization will occur once the skin incision has been closed. In cases of bilateral groin incision the right groin will be randomized and the left allocated the other arm of the trial. The surgeons performing the procedure will not have prior knowledge of the type of dressing to applied at the end of the operation. The operating room personnel and the investigators evaluating the outcome will not be blinded to the intervention.

Sample size calculation: Based on previous studies[12, 19] the incidence of postoperative groin SSI is 30-35%. Recent studies using NPWT have shown a reduction in incidence of 50% or more.[17, 18] Using a sample size calculator (Sealed Envelope Ltd., Clerkenwell Workshops, London EC1R 0AT, UK) for binary outcome superiority trials with an alpha level of 0.05 for 80% power and an estimated incidence reduction from 30% to 10% using NPWT a sample size of 59 patients in each arm and a total sample size of 118 is required.

Ethical considerations: The study will be performed in accordance with the declaration of Helsinki as revised at the 64th WMA General Assembly in Fortaleza, Brazil, October 2013. Approval of the study protocol has been obtained from the Regional Ethics Committee.

Informed consent Prior to enrolment all patients will receive written and oral information about the study. The patients will only be included after obtaining written and informed consent.

References:

1.Vogel TR, Dombrovskiy VY, Carson JL, Haser PB, Lowry SF, Graham AM. Infectious complications after elective vascular surgical procedures. Journal of vascular surgery. Jan 2010;51(1):122-129; discussion 129-130.2. Kent KC, Bartek S, Kuntz KM, Anninos E, Skillman JJ.

2.Prospective study of wound complications in continuous infrainguinal incisions after lower limb arterial reconstruction: incidence, risk factors, and cost. Surgery. Apr 1996;119(4):378-3833. de Lissovoy G, Fraeman K, Hutchins V, Murphy D, Song D, Vaughn BB.

3.Surgical site infection: incidence and impact on hospital utilization and treatment costs. American journal of infection control. Jun 2009;37(5):387-397.

4. Kuy S, Dua A, Desai S, et al. Surgical site infections after lower extremity revascularization procedures involving groin incisions. *Annals of vascular surgery.* Jan 2014;28(1):53-58.
5. Krizek TJ, Robson MC. Evolution of quantitative bacteriology in wound management. American journal of surgery. Nov 1975;130(5):579-584.
6. Arbeit RD, Dunn RM. Expression of capsular polysaccharide during experimental focal infection with Staphylococcus aureus. *The Journal of infectious diseases.* Dec 1987;156(6):947-952.
7. Garibaldi RA, Cushing D, Lerer T. Risk factors for postoperative infection. The Americanjournal of medicine. Sep 16 1991;91(3B):158S-163S.
8. Vriesendorp TM, Morelis QJ, Devries JH, Legemate DA, Hoekstra JB. Early post-operative glucose levels are an independent risk factor for infection after peripheral vascular surgery. A retrospective study. *European journal of vascular and endovascular surgery : the official journal of the European Society for Vascular Surgery.* Nov 2004;28(5):520-525.
9. Chang JK, Calligaro KD, Ryan S, Runyan D, Dougherty MJ, Stern JJ. Risk factors associated with infection of lower extremity revascularization: analysis of 365 procedures performed at a teaching hospital. *Annals of vascular surgery. Jan* 2003;17(1):91-96.
10. Anderson DJ. Surgical site infections. Infectious disease clinics of North America. Mar 2011;25(1):135-153.
11. Lee ES, Santilli SM, Olson MM, Kuskowski MA, Lee JT. Wound infection after infrainguinal bypass operations: multivariate analysis of putative risk factors. Surgical infections. Winter 2000;1(4):257-263.
12. Health Protection Agency. (2012) English National Point Prevalence Survey on Healthcare Associated Infections and Antimicrobial Use, 2011: Preliminary data. 2011.
13. Cater JE, van der Linden J. Simulation of carbon dioxide insufflation via a diffuser in an open surgical wound model. *Medical engineering & physics.* Jan 2015;37(1):121-125.
14. Turtiainen J, Saimanen EI, Makinen KT, et al. Effect of triclosan-coated sutures on the incidence of surgical wound infection after lower limb revascularization surgery: a randomized controlled trial. *World journal of surgery.* Oct 2012;36(10):2528-2534.
15. Dosluoglu HH, Loghmanee C, Lall P, Cherr GS, Harris LM, Dryjski ML. Management of early (<30 day) vascular groin infections using vacuum-assisted closure alone without muscle flap coverage in a consecutive patient series. *Journal of vascular surgery.* May 2010;51(5):1160-1166.
16. Weed T, Ratliff C, Drake DB. Quantifying bacterial bioburden during negative pressure wound therapy: does the wound VAC enhance bacterial clearance? *Annals of plastic surgery.* Mar 2004;52(3):276-279; discussion 279-280.
17. Matatov T, Reddy KN, Doucet LD, Zhao CX, Zhang WW. Experience with a new negative pressure incision management system in prevention of groin wound infection in vascular surgery patients. *J Vasc Surg*. 2013 Mar;57(3):791-5. doi: 10.1016/j.jvs.2012.09.037.
18. Hyldig N, Birke-Sorensen H, Kruse M, Vinter C, Joergensen JS, Sorensen JA, Mogensen O, Lamont RF, Bille C. Meta-analysis of negative-pressure wound therapy for closed surgical incisions. *Br J Surg.* 2016 Apr;103(5):477-86.
19. Daryapeyma A, Hammar U, Wahlgren CM. Incidence of Healthcare Associated Infections After Lower Extremity Revascularization Using Antibiotic Treatment as a Marker. *Eur J Vasc Endovasc Surg.* 2016 May;51(5):690-5.
